# Establishment of an integrated automated embryonic manipulation system for producing genetically modified mice

**DOI:** 10.1038/s41598-021-91148-9

**Published:** 2021-06-03

**Authors:** Tomoo Eto, Hiroki Ueda, Ryoji Ito, Tsukasa Takahashi, Toshiaki Watanabe, Motohito Goto, Yusuke Sotomaru, Nobuaki Tanaka, Riichi Takahashi

**Affiliations:** 1 Central Institute for Experimental Animals, 3-25-12, Tonomachi, Kawasaki-ku, Kawasaki, 210-0821 Japan; 2 New Field Products Development Center, NSK Ltd., 1-5-50, Kugenuma Shinmei Fujisawa-shi, Kanagawa, 25-8501 Japan; 3 Natural Science Center for Basic Research and Development, Hiroshima University, 1-2-3, Kasumi, Hiroshima, Hiroshima, 734-8551 Japan

**Keywords:** Biological techniques, Biotechnology, Developmental biology, Molecular biology

## Abstract

Genetically modified mice are commonly used in biologic, medical, and drug discovery research, but conventional microinjection methods used for genetic modification require extensive training and practical experience. Here we present a fully automated system for microinjection into the pronucleus to facilitate genetic modification. We first developed software that automatically controls the microinjection system hardware. The software permits automatic rotation of the zygote to move the pronucleus to the injection pipette insertion position. We also developed software that recognizes the pronucleus in 3-dimensional coordinates so that the injection pipette can be automatically inserted into the pronucleus, and achieved a 94% insertion rate by linking the 2 pieces of software. Next, we determined the optimal solution injection conditions (30 hPa, 0.8–2.0 s) by examining the survival rate of injected zygotes. Finally, we produced transgenic (traditional DNA injection and piggyBac Transposon system) and knock-in (genomic editing) mice using our newly developed Integrated Automated Embryo Manipulation System (IAEMS). We propose that the IAEMS will simplify highly reproducible pronuclear stage zygote microinjection procedures.

## Introduction

Genetically modified mice are widely used in broad areas of biologic, medical, and drug discovery research^1–4^. Genetic modification of mammals was first reported in 1980 using mice^5^. Since then, several methods, such as transposon methods^6,7^, have been developed to improve the efficiency of producing transgenic mice. The advent of genome-editing technologies has additionally revolutionized the generation of genetically engineered mice. For example, gene targeting^8^ and knock-in^9,10^ methods previously performed in embryonic stem cells are now performed in zygotes via genome editing. Various methods are used for genetic modification in mice, such as manual injection of a solution into the pronucleus of the mouse zygote. This method was used to perform the first genetic modification^5^ of mammals and is widely applied in many laboratories. Current microinjection techniques, however, require extensive training because the hardware must be manually operated in a complicated manner, even when injecting a solution into only 1 zygote^11^. In addition, mastering microinjection techniques requires years of experience^12^. Although electric injectors and electric joysticks have been developed to assist with the manual operations^13,14^, the most critical steps of the microinjection still depend on manual operation (i.e., determining the zygote holding site, adjusting the focus on the zygote, selecting the injection site, and injecting the correct volume). Therefore, microinjection is a painstaking technique that is applied to 1 zygote at a time by only highly skilled microinjectionists^12^.

In the present study, we designed a fully automated method for injecting a solution into the zygote pronucleus that makes genetic modifications by microinjection easy and highly reproducible. First, we identified the sequence of actions required for fully automated operation and developed hardware that combines multiple machines. We then designed new software to operate the hardware. Finally, we developed the Integrated Automated Embryonic Manipulation System (IAEMS), which integrates the newly developed hardware and software, and demonstrated the usefulness of the IAEMS for producing genetically modified mice.

## Results

### Sequences, hardware, and basic software for automated injection

**Figure 1:**
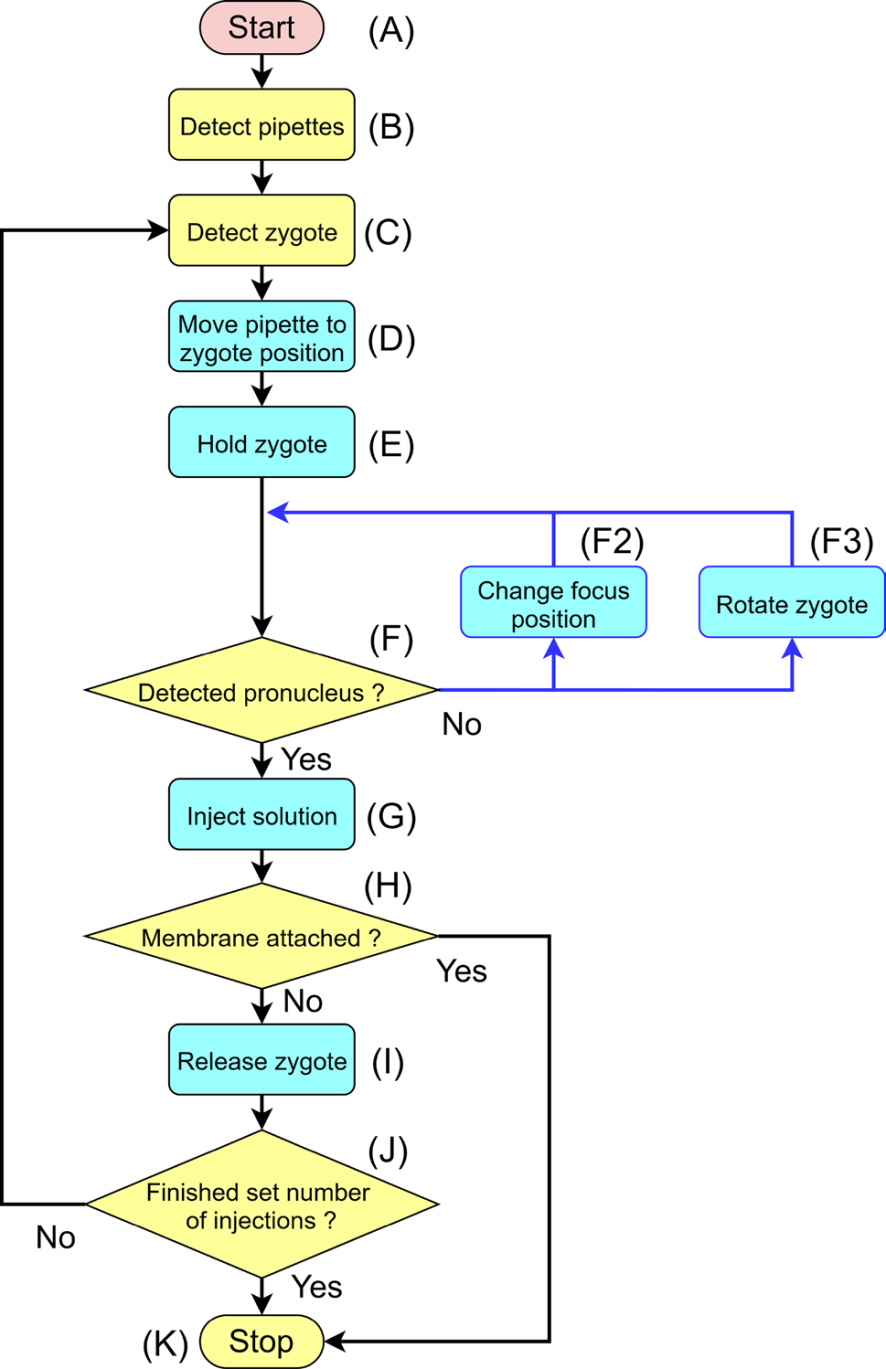
Sequence required for fully automated solution injection. The sequence involves detection of the injection target and judgment of the work at each step (yellow), and then execution of the work (blue). (**A**) Command the controller (Computer) to start the fully automated operation. (**B**) Detect the coordinates of the current positions of the pipettes. (**C**) Detect the zygote position. (**D**) Move the pipette to the zygote position. (**E**) Hold the zygote in the holding pipette and release the zygote once to adjust the holding pressure. (**F**) Detect the current position of the pronucleus. If the pronucleus cannot be detected, change the height of the holding pipette while holding the zygote (**F2**), and then re-detect the pronucleus (**F**). If the pronucleus is not detected after performing sequence **F2** multiple times, use an injection pipette to rotate the zygote vertically (**F3**) and then re-detect the pronucleus (**F**). (**G**) Insert the injection pipette into the pronucleus. (**H**) If, after injection of the solution, the nuclear membrane adheres to the injection pipette and does not come off, then stop the procedure(**K**). Remove the nuclear membrane from the injection pipette and restart (**A**). (**I**) Move the zygote to the release area and release the zygote (Fig. S3). (**J**) Check whether the injection has been performed on all zygotes. If an injection zygote remains that has not been injected, return to sequence **C** and continue injecting. (**K**) After injecting the solution into all available zygotes, stop the fully automated injection process.

**Figure 2:**
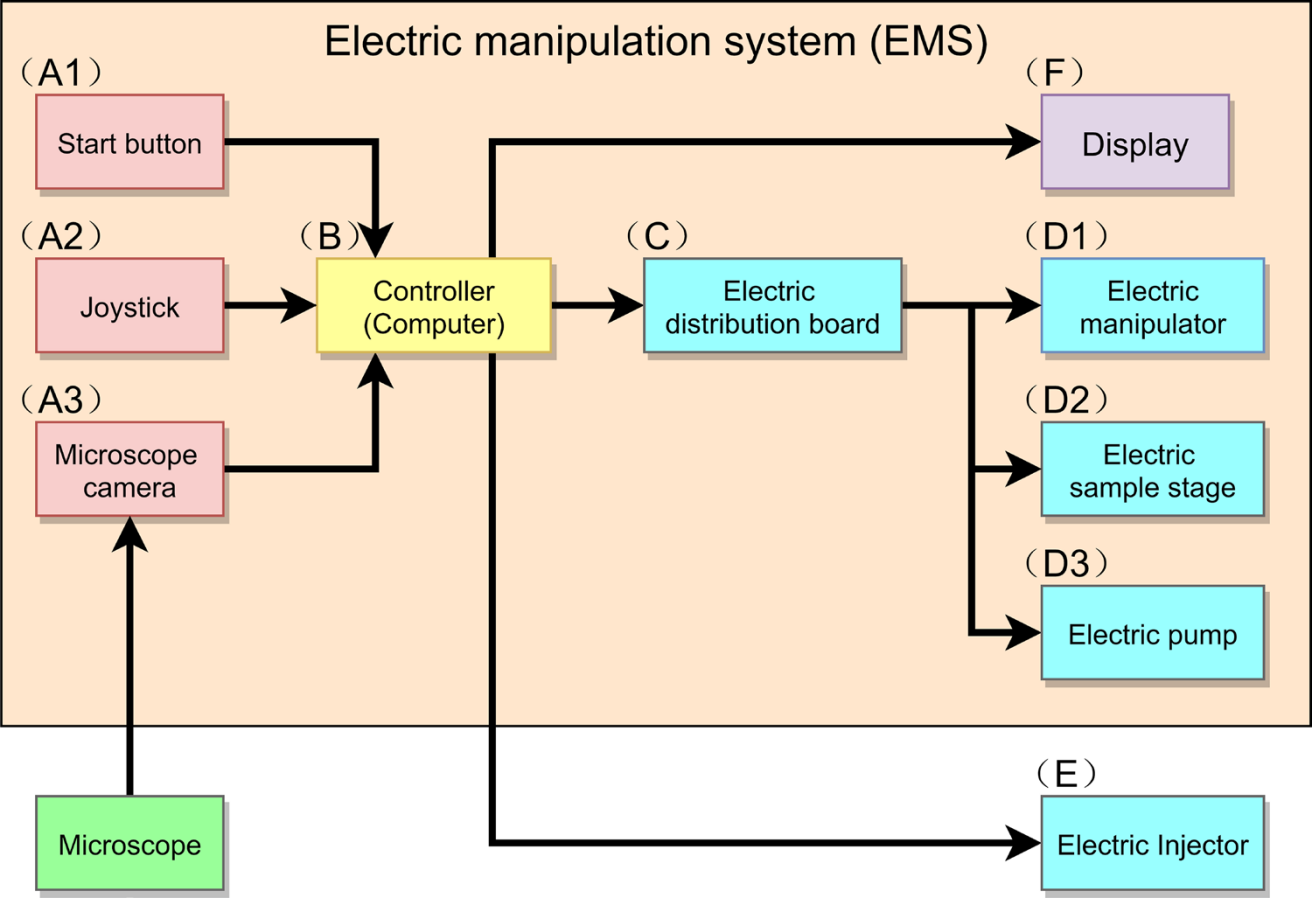
Hardware. The hardware was created by connecting an electric injector to an electric manipulation system, and comprises 3 main elements: input device (red), controller (yellow), and output device (blue). In the hardware, when the signal is input from the outside (**A**), the controller analyzes the signal (**B**), the electric distribution board controls multiple machines according to the analyzed data (**C**) and operates multiple electric machines (**D**,**E**). (**D**) The electric manipulator moves the pipettes 3-dimensionally (**D1**), the electric sample stage moves the dish containing the zygotes 2-dimensionally (**D2**), and the electric pump increases or decreases the holding pressure in the holding pipette (**D3**). (**E**) The electric injector injects the solution by applying the injection pressure for the amount of time that was previously set in the controller. Only the electric injector (**E**) operates with a serial communication from the controller (**B**). In a fully automated operation, when the start button (**A1)** signal is input to the controller, multiple machines (**D,E**) operate electrically based on the microscope image acquired by the microscope camera (**A3**). In manual mode, the operator manually operates the joystick (**A2**) while looking at the display (**F**) to enter operational commands into the controller (**B**) to electrically operate multiple machines (manipulator (**D1**), sample stage (**D2**), and pump (**D3**)). In addition, in solution injection, the operator manually inputs the operation signal directly to the electric injector (**E**).

In this study, we first designed the sequence required to achieve fully automated solution injection (Fig. 1). The sequence comprises a series of operations, such as immobilizing the zygote, focusing on the pronucleus, injecting the solution, and releasing the zygote. Thus, injection is performed automatically after manually transferring several zygotes to the injection medium drop. Next, to realize the sequence, we developed hardware that incorporates the electric manipulation system (EMS) and electric injector that we previously developed into a microscope (Fig. 2, Fig. S1). Currently existing manual microinjection systems are equipped with a manipulator that moves the pipette in 3-dimensions, a sample stage that moves the zygote in 2 dimensions, and a pump for solution injection. For the microinjection system to be fully automatic, all devices must be driven electrically and in tandem. The currently existing manual microinjection devices cannot support full automation; for automation, all devices must be powered and the EMS is used to control the devices with the controller and electric distribution board. Further, for accuracy, each device needs to make the fine movements required to perform microinjection; all EMS devices have the ability to perform the necessary fine movements (Table S1). In addition, the software installed in the EMS automatically detects the zygote position (Fig. S2), moves the pipette near the non-injected zygote, and moves the injected zygote to the release area (Fig. S3).

### Automated pipette insertion into the pronucleus

**Figure 3:**
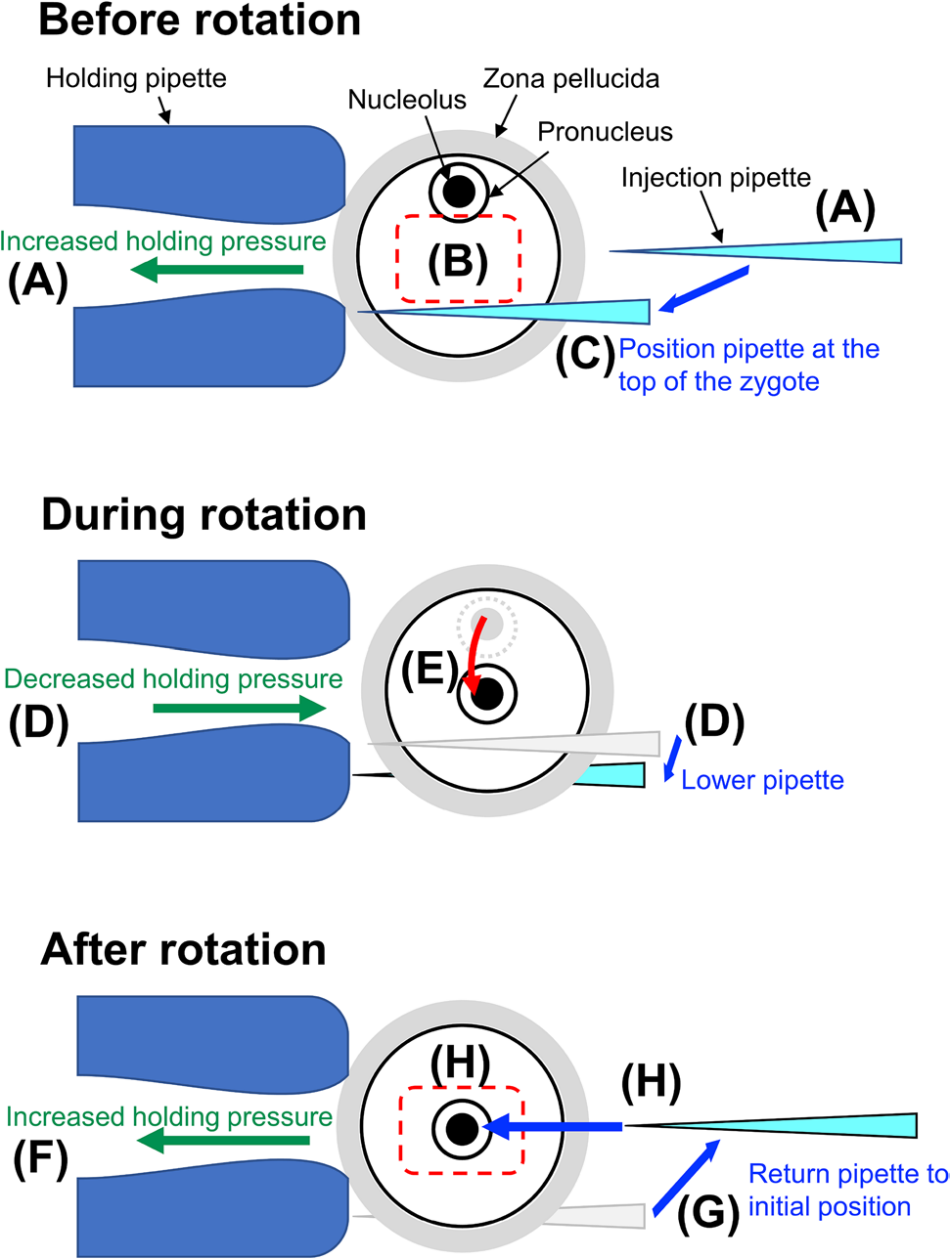
Procedure to fully rotate the zygote to move the pronucleus to the injection pipette insertion position. (**A**) Before rotation, the zygote is held in the holding pipette under a state of increased holding pressure. The injection pipette is held stationary at the middle height position of the zygote. (**B**) If the pronucleus inside the zygote is outside the range (red dotted square) where automated solution injection is possible. (**C**) The injection pipette automatically moves to the upper part of the zygote opposite the pronucleus. (**D**) Due to the decreased holding pressure, the zygote disengages from the holding pipette, and immediately lowering the injection pipette causes the zygote to rotate. (**E**) With the rotation, the pronucleus moves from the corner of the zygote to the center (red arrow). (**F**) Immediately after rotation, the holding pressure is increased to hold the zygote. (**G**) The injection pipette is returned to its initial position (**A**). (**H**) When the pronucleus is within the range for automated solution injection (red dotted square), the injection pipette is inserted into the pronucleus.

**Figure 4:**
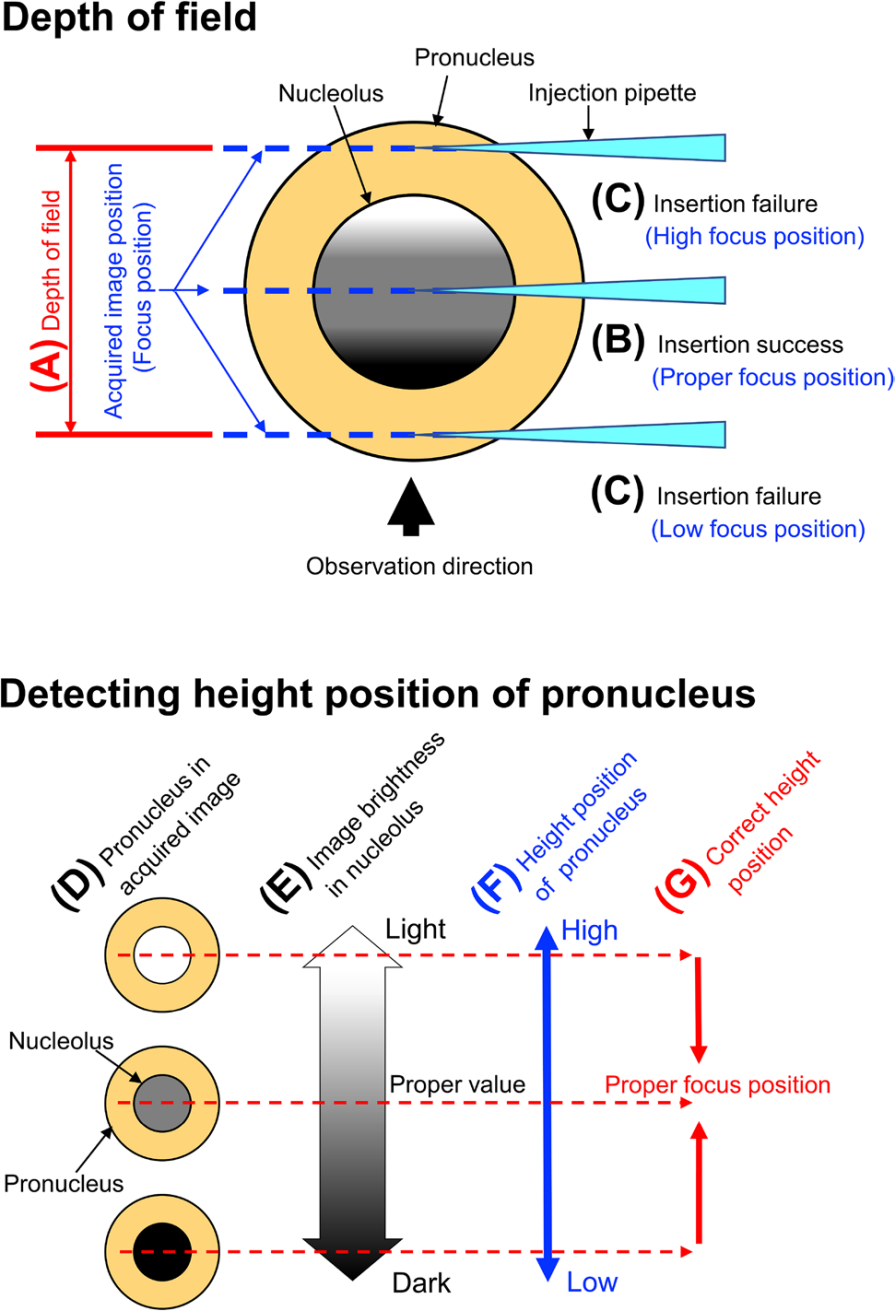
Procedure for detecting the height position of the center of the pronucleus during automated solution injection. (**A**) Under microscope observation, the depth of field (red arrow) varies, so the focus position of the acquired image and the height position of the pronucleus may be different each time. (**B**) If the nucleolus is identified at the proper focus position for pipette insertion, the pipette can be inserted. (**C**) On the other hand, if there is a difference in height between the focus position and pronucleus position, the nuclear membrane will not break and the pipette cannot be inserted. To ensure that the pipette is placed at the proper position, it is necessary to detect and correct the height position of the pronucleus. (**F**) From the pronucleus in the acquired image (**D**), obtain the brightness value of the nucleolus (**E**), and detect the height position of the pronucleus. (**G**) If a height position other than the proper focus position is detected, correct the proper focus position and insert the pipette.

In conventional manual injection procedures, the operator visually confirms the position of the pronucleus, moves the pronucleus to a position where the pipette can be inserted by rotating the zygote, and then inserts the pipette into the pronucleus. To our knowledge, no software exists to perform this operation automatically, so we developed 2 new software programs in LabVIEW. First, we developed software that automatically rotates the zygote to correctly position the pronucleus for insertion of the injection pipette (Fig. 3, Movie S1). Rotation of the zygote is achieved by changing the holding pressure of the holding pipette and moving the zygote to contact the injection pipette. We then developed software that recognizes the pronucleus location with 2-dimensional (2D) coordinates so that the injection pipette can be automatically inserted into the nucleus. With this software, however, the success rate of pipette insertion into the pronucleus was only 70.0% (n = 50). Therefore, we next developed software that recognizes the pronucleus location with 3-dimensional (3D) coordinates, which has the ability to correct the height position of the pipette. Using this software, the success rate of automated pipette insertion was significantly improved to 94% (n = 50) (Fig. 4). In subsequent experiments, the zygote rotation software was combined with the 3D software to automatically insert an injection pipette into the nucleus.

### Establishment of automated pronuclear injection conditions

Injecting a large volume of solution into the pronucleus adversely affects the zygote^11^. Therefore, it is important to determine the proper settings for both the injection pressure and injection time, which determine the injection volume^15^. In this experiment, optimization of the injection pressure and injection time was aimed at injecting as much volume as possible without decreasing the zygote survival rate. Zygote survival was determined by observing the oocyte morphology 1 h after injection.

**Figure 5:**
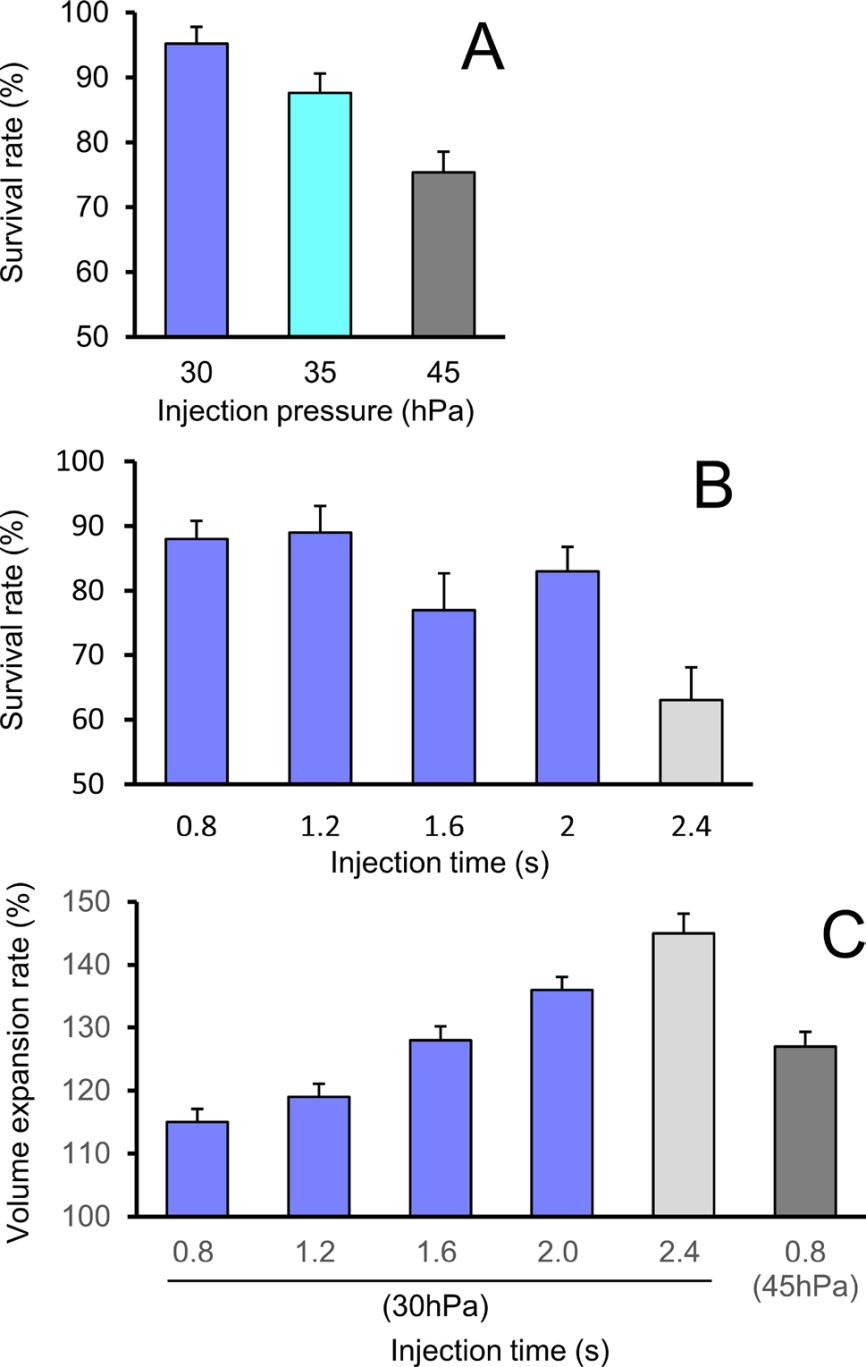
Injection pressure and injection duration influence the zygote survival rate and expansion rate. (**A**) The infusion time was fixed at 0.8 s and zygote survival was examined at infusion pressures of 30, 35, and 45 hPa. (**B**) With a constant pressure of 30 hPa, zygote survival was examined at injection times of 0.8, 1.2, 1.6, 2.0, and 2.4 s. (**C**) The pronuclear volume expansion rate when injected at the indicated pressure.

First, the appropriate injection pressure was determined. The injection time was fixed at 0.8 s and the solution was injected at 30, 35, or 45 hPa (n = 188, 191, and 200 zygotes, respectively, for each injection pressure). The zygote survival rate was nearly 100% at 30 hPa (Fig. 5A), but the survival rate decreased significantly when the injection pressure exceeded 35 hPa. We then examined the solution injection time. The injection pressure was fixed at 30 hPa, and the solution was injected over 0.8, 1.2, 1.6, 2.0, or 2.4 s (n = 100/injection time). Zygote viability after the solution injection decreased significantly when the injection time exceeded 2.0 s (Fig. 5B). In this range, the volume of the pronucleus expanded linearly with the injection time (Fig. 5C). The volume achieved at 45 hPa for 0.8 s was similar to that at 30 hPa for 1.6 s, suggesting that the injected volume is not simply determined by the product of the injection time and injection pressure. In the following experiments, the injection pressure was fixed to 30 hPa, because the volume of the injection solution is finely adjustable by changing the time.

### Production of genetically modified mice

**Figure 6:**
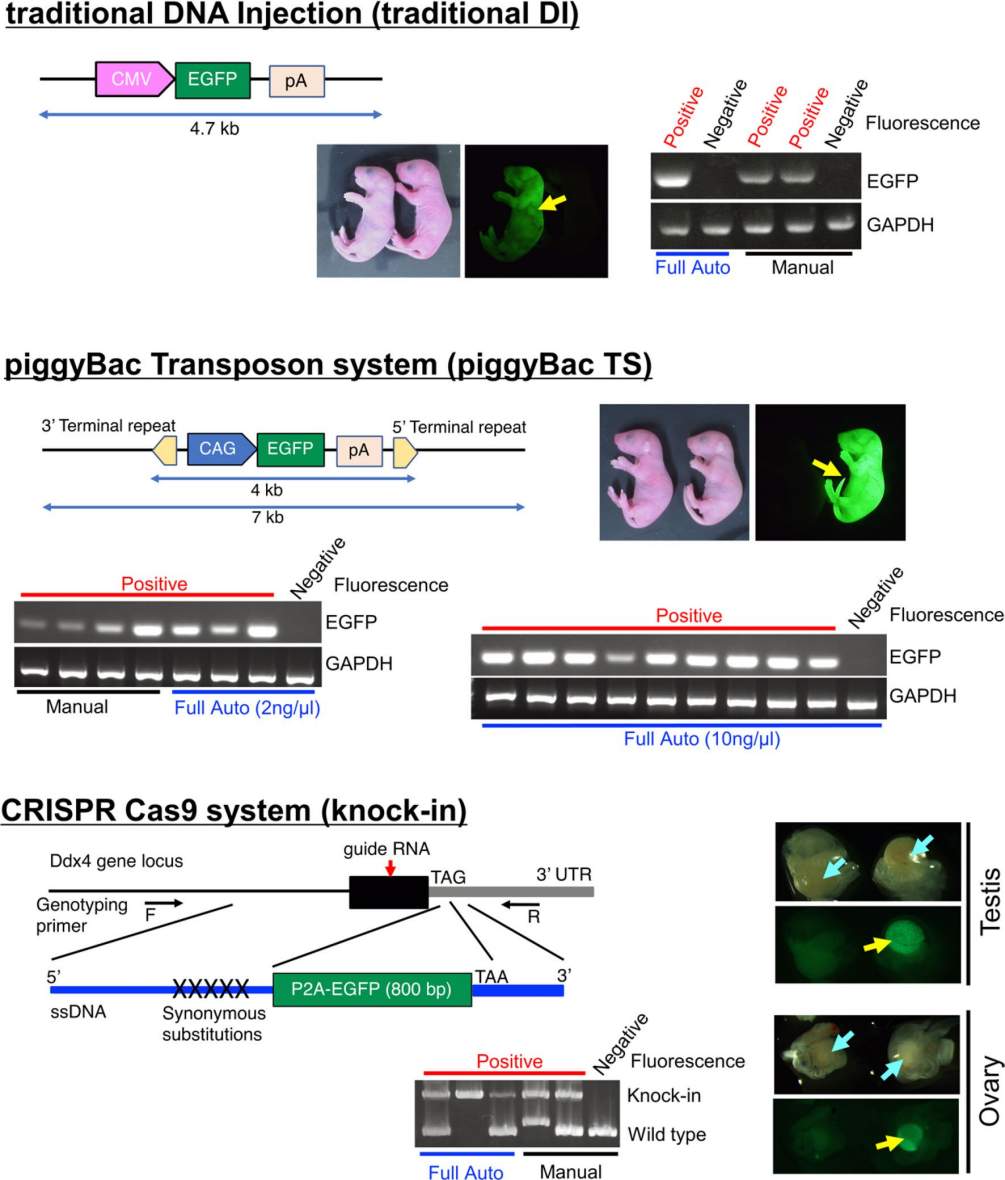
EGFP expression and gene transfer in genetically modified mice produced by 3 different methods. Different DNA constructs were used to produce the 3 types of genetically modified mice. Mice produced by fully automated or manual injection were examined for EGFP expression by UV irradiation and for gene modification by genomic DNA PCR. Yellow arrows indicate typical examples of testes and ovaries containing germ cells with confirmed EGFP fluorescence. Blue arrows indicate typical examples of ovaries or testes before UV irradiation. EGFP fluorescence-positive mice were always positive on PCR examination.

When producing genetically modified mice by injecting a solution, the type and concentration of the nucleic acids and proteins added to the solution differ depending on the method. Therefore, it is necessary to fully automatically inject multiple types of solutions into zygotes to confirm whether genetically modified mice can be produced in each case. In this study, we produced genetically modified mice using 3 different methods: traditional DNA injection (traditional DI) using linearized plasmid DNA; a piggyBac Transposon system (piggyBac TS) using circular plasmid DNA and transposase mRNA; and a CRISPR-Cas9 system (knock-in) using single-stranded DNA, guide RNA, and Cas9 nuclease protein (Fig. 6). In all of the experiments, the mouse was judged to be genetically modified when positive results were obtained in both enhanced green fluorescent protein (EGFP) fluorescence analyses and genotyping polymerase chain reaction (PCR) analyses (Fig. 6, Fig. S4). In contrast to the ubiquitous expression of EGFP in mice generated by traditional DI and piggyBac TS experiments, the reporter gene expression was restricted to germ cells in mice generated by knock-in, because EGFP is knocked into the germ cell-specific gene (Ddx4) locus.

**Figure 7:**
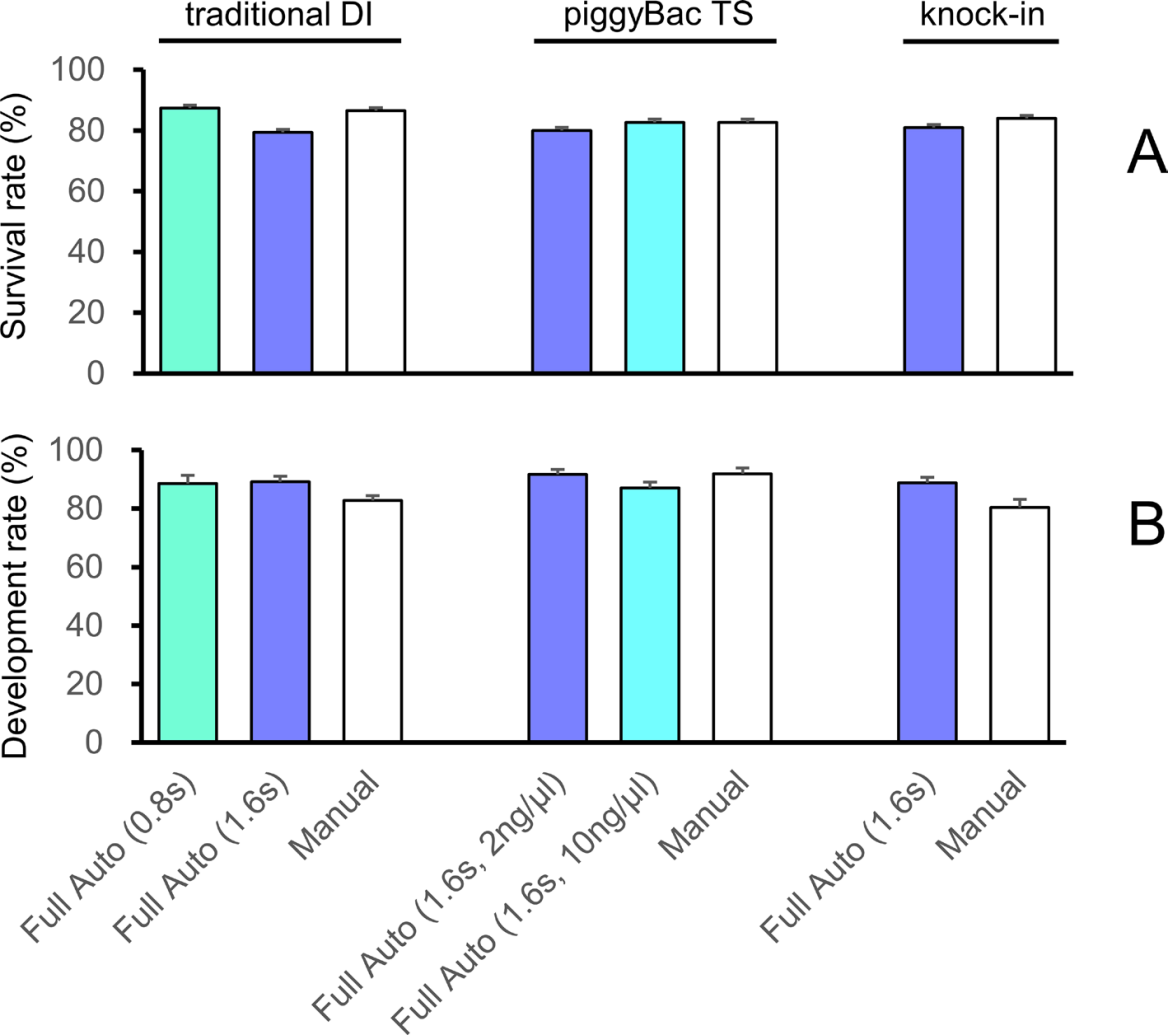
Survival and in vitro development of zygotes injected into the pronucleus with solutions used for 3 different genetic modifications. In the fully automated solution injection, all injection pressures were fixed at 30 hPa, and the injection time was 0.8 or 1.6 s for traditional DNA injection (traditional DI), and 1.6 s for the piggyBac Transposon system (piggyBac TS) and CRISPR-Cas9 system (knock-in). In addition, the piggyBac TS used solutions with a DNA concentration of 2 or 10 ng/µl. The survival rate (**A**) and development rate to a 2-cell stage embryo (**B**) after solution injection did not differ significantly among groups with fully automated and manual injections.

First, a traditional DI experiment was performed under 3 conditions (automated injection for 0.8 s, automated injection for 1.6 s, and manual injection). We injected 151 (0.8 s), 199 (1.6 s), and 148 (manual) embryos using plasmid DNA at a concentration of 1.5 ng/µl. The survival rate of the zygotes was 87.4%, 79.4%, and 86.5%, respectively, and the in vitro development rate to 2-cell embryos was 88.6%, 89.2%, and 82.8%, respectively (Fig. 7, Table S2). The results did not differ significantly between the automated and manual injection methods with regard to survival or development rates. Importantly, the percentage of pups obtained from transplanted embryos injected automatically was 19.7% (0.8 s) and 19.1% (1.6 s), and tended to be higher than the percentage of pups obtained from manual injection (5.7%; Table 1). The number of genetically modified mice obtained from embryos injected using the automated injection system, however, was 0 (0.8 s) and 1 (1.6 s), and manual injection generated 2 genetically modified mice. Thus, in these conditions, the manual injection produced genetically modified animals more efficiently than the automated injection method. The higher survival rate and lower modification rate in the automated injection method suggests that the amount of injected DNA in the automated injection should be increased to achieve maximum efficiency. Next, the piggyBac TS experiment was conducted using automated (injection time 1.6 s) and manual injection. Two different plasmid DNA concentrations were tested for automated injection (2 and 10 ng/µl). In the manual condition, 2 ng/µl plasmid DNA was injected. The survival rate (2 ng/µl automated; 80.0%, 10 ng/µl automated; 82.7%, manual; 82.7%) and development rate to 2-cell stage embryos (2 ng/µl automated; 91.7%, 10 ng/µl automated; 87.1%, manual; 91.9%) did not differ significantly among the 3 conditions (Fig. 7). In addition, using 2 ng/µl DNA, the fully automated and manual results were similar (2.7% and 3.5%, respectively; Table 1). Furthermore, we demonstrated that even piggyBac^16^ transgenesis can be reproduced fully automatically because the transgenic mouse production rate improved when the DNA concentration was increased (10 ng/µl; 8.3%). Experimental results of traditional DI and piggyBac TS suggest that transgenic mice can be produced even with fully automated solution injection.

**Table 1:** Offspring and genetic modification of zygotes injected into the pronucleus with solutions used for 3 different genetic modifications.

Genetic modification method	Injection method	Injection time (s)	DNA concentration (ng/µl)	Number of embryos transferred	Offspring (%)	Genetically modified (%)
Traditional DI	Full auto	0.8	1.5	117	23 (19.7)	0 (0.0)
		1.6	1.5	141	27 (19.1)	1 (0.7)
	Manual	–	1.5	106	6 ( 5.7)	2 (1.9)
piggyBac TS	Full auto	1.6	2	110	20 (18.2)	3 (2.7)
		1.6	10	108	24 (22.2)	9 (8.3)
	Manual	–	2	114	26 (22.8)	4 (3.5)
Knock-in	Full auto	1.6	2	143	37 (25.9)	3 (2.1)
	Manual	–	2	135	15 (11.1)	2 (1.5)

The Offspring and Genetically modified percentages were calculated using the number of embryos transferred as the denominator.

In the knock-in experiment, automated solution injection was performed in 1.6 s using 2 ng/µl of single-stranded DNA. The automated injection and manual injection methods were not significantly different with regard to the survival rate (80.9% and 84.0%, respectively) and the development rate to 2-cell stage embryos (88.8% and 80.4%, respectively; Fig. 7). In addition, the offspring production rate was higher for the automated injection method (25.9%) than the manual injection method (11.1%), and the gene modification rates were comparable (2.1 and 1.5%, respectively; Table 1). These results suggested that knock-in mice produced by genome editing can be produced with fully automated solution injection.

## Discussion

This study reports for the first time (1) automated nuclear injection and (2) production of genetically modified animals using fully automated injection. Rapid production of new genetically modified mice will accelerate medical, pharmaceutical, and biology research. Multiple methods currently exist for genetically modifying zygotes. One genetic modification method is electroporation^17^, but the generation of transgenic mice is difficult using traditional DI^5^, piggyBac TS^18^, and artificial chromosomes (YACs, BACs, and PACs)^19^. Furthermore, in genome editing, gene knock-in tends to be a less efficient method for producing genetically modified mice than gene knock-out^20^. Although a method has been developed for injecting DNA solutions into the cytoplasm, the types of genes that can be introduced are limited compared with those that can be directly injected into the pronucleus^18,20^. Injecting the solution into the pronucleus is considered suitable for various genetic modifications, but the conventional method is a complicated procedure that requires highly skilled microinjectionists. The conventional method cannot likely be sufficiently modified to more rapidly provide genetically modified mice. Therefore, we developed a fully automated injection system for producing genetically modified mice.

We considered the injection site of the solution before designing the sequence for the fully automated solution injection. The potential sites for solution injection into zygotes are the cytoplasm^21–23^ and pronucleus, but in the first reports of traditional DI^5^, piggyBac TS^18^, and knock-in genome editing^9,10^, the solution was injected into the pronucleus. In addition, in traditional DI, intracellular injection significantly reduces the efficiency of transgenic mouse production compared with intranuclear injection^20^. Furthermore, in genome editing using CRISPR Cas9, the concentration of vector DNA contained in the injection solution must be 20 times higher when injected into the cytoplasm than when injected into the pronucleus^24^. Therefore, in the present study, the solution was injected into the pronucleus.

We next considered how to immobilize the zygote. The injection pipette cannot be inserted into the pronucleus without immobilizing the zygote^11,15^. In methods utilizing semi-automated solution injection into the cytoplasm, the injection chamber applies suction to immobilize the zygote^21–23^. With this method, however, it is difficult to insert an injection pipette into the pronucleus because the zygote cannot be freely rotated to optimize the position of the pronucleus relative to the injection pipette. Therefore, to fully automate the procedure, we used the same pipette system used in the manual injection method^11,15^ (Fig. 3, Movie S1).

For manual injection, an injection pipette is appropriately inserted into the pronucleus by a microinjectionist, which requires many years of experience. Because it is difficult to automatically reproduce extensive experience, however, we developed software to determine the optimal insertion site. For automated insertion of the injection pipette into the pronucleus, the controller must accurately recognize the position of the pronucleus in the cytoplasm. Therefore, we also developed software that recognizes the pronucleus in 2 dimensions utilizing a generalized Hough transform^25^ of the obtained digital image. To accurately detect an object using the generalized Hough transform, the outline of the object in the image must be sharp. In this study, nucleoli whose contours were clearly confirmed in many pronuclei were targeted for acquiring the position coordinates. The injection success rate using the 2D system, however, was only 70%. In a digital image, while the 2D coordinates of the nucleolus can be obtained, the 3D solid coordinates, including the height direction, cannot be obtained. When observing a 3D structure with an inverted microscope, the depth of field that appears to be in focus on the object varies (Fig. 4). Therefore, even if the nucleolus is displayed in the acquired image, the focal position and the actual height of the nucleolus may not match. It is likely that the height of the actual nucleolus will not match that of the injection pipette and thus the injection pipette will miss the pronucleus. Therefore, in addition to the 2D coordinates obtained by the Hough transform, we developed software that recognizes the pronucleus in 3 dimensions by correcting the distance in the height direction between the position of the nucleolus and the focal position from the image brightness value of the nucleolus in the image (Fig. 4). Using the 3D coordinate software, we obtained an insertion success rate of 94%, which was significantly higher than that using the 2D coordinate software. In the following experiments, we therefore used the software that provides the pronucleus location in 3D coordinates.

When manually injecting a solution into the pronucleus, the nucleus tends to swell within 1 s at an injection pressure of 30–40 hPa^20^. Therefore, to examine the injection pressure, the injection time was fixed at 0.8 s and the pressure was increased stepwise from 30 to 45 hPa. The results of the experiment indicated that the survival rate was the same at 30 hPa and 35 hPa, and significantly decreased at 45 hPa. Because increasing the injection pressure above 35 hPa decreased the survival rate, the injection pressure was fixed at 30 hPa and the injection time was examined. Survival rates were similar for infusion times ranging from 0.8 to 2.0 s, but significantly decreased at 2.4 s. As shown in Fig. 5, a 2.0-s injection at 30 hPa, resulting in an expansion of the pronucleus to 136% of the original volume, had little effect on the zygote survival rate. Although the expansion rates at 30 hPa for 1.6 s and 45 hPa for 0.8 s were equivalent, the survival rate at 45 hPa and 0.8 s was lower. If the injection pressure is too high, the zygote may be damaged even if the injection amount is equivalent to that produced at a lower injection pressure^20^. These findings suggested that performing the solution injection at the lowest possible pressure over a longer time should improve the survival rate. Therefore, the injection pressure was fixed at 30 hPa and the injection time was extended stepwise from 0.8 s in the experimental production of genetically modified mice.

The automated and manual injection methods did not differ significantly with respect to zygote survival and development to 2-cell stage embryos in the traditional DI, piggyBac TS, and knock-in experiments after injecting a solution at 30 hPa over 1.6 s (Fig. 7). The solutions used in each experiment had different contents. Thus, injection settings rather than the injection method (i.e., automated or manual) or injected solution were likely the major determinants of zygote survival and development rates. In traditional DI, only a small number of transgenic mice was obtained using a 1.5-ng/µl DNA solution, both manually and automatically. In traditional DI, however, the gene modification rate fluctuates depending on the increase or decrease in the DNA concentration^20^. In the future, we would like to evaluate the effect of the DNA concentration on the production of transgenic mice by traditional DI using fully automated solution infusion.

On the basis of the experiments using the IAEMS, we found that genetically modified mice can be produced even by fully automated injection of multiple types of solutions (Table 1). Several methods of automatically injecting a solution into zygotes have been reported. For example, while automated injection into the cytoplasm has been reported in zebrafish^21^ and drosophila^22^, genetic modification has not been achieved. In mammals, automated injection into the cytoplasm has been reported in mice^23^, but its effect on genetic modification was not examined.

Through this research, we were able to develop an IAEMS that can easily and reproducibly microinject solutions into the pronucleus of a zygote without extensive training or experience. We believe that the use of the IAEMS will make it easier to produce genetically modified mice for research in biology, medicine, and pharmaceutical sciences. In the next step, we will apply the IAEMS to intracytoplasmic sperm injection^26^, which is used in fertility treatment. The development of software and hardware for automated micromanipulation will be an important research topic for future progress in the natural sciences for humans and animals.

## Methods

### Animals

Jcl:BDF1 mice were used to collect zygotes at the pronuclear stage (female: 8–16 weeks old, male: 12–20 weeks old). Jcl:MCH(ICR) female mice 10–16 weeks of age were used as recipients for embryo transfer. All mice were purchased from CLEA Japan Inc. (Tokyo, Japan). The mice were reared under the following conditions: room temperature 22 °C ± 0.5 °C, humidity 55% ± 5%; lights on 08:00–20:00. Food (CA-1; CLEA Japan) and water were provided ad libitum.

All animal experiments were approved by the Animal Committee of Central Institute for Experimental Animals, and the study was performed in accordance with ARRIVE guidelines (Animal Research: Reporting of In Vivo Experiments) and with Central Institute for Experimental Animals guidelines.

### Collection, cryopreservation, and embryo transfer of zygotes

Zygotes were collected by in vitro fertilization^27^. Approximately 8 h after insemination by the sperm, the pronuclear zygotes were cryopreserved using the vitrification method of Eto et al.^27^. Vitrified pronuclear oocytes were used for injection of the solution 1 h after warming. The pronuclear oocytes injected with the solution were cultured in vitro in KSOM medium^28^, and 2-cell stage embryos were transplanted to recipient females in the morning of the next day (10:00–12:00). The solutions used for these embryo manipulations were purchased from ARK Resource (Kumamoto, Japan).

### Solution Injection into pronuclear zygotes

The hardware was made by mounting an EMS (XY-MC0202-701–001; NSK Ltd., Tokyo Japan) and an electric injector (Femtojet 4i, Eppendorf AG, Hamburg, Germany) onto a microscope (ECLIPSE Ti2-U; Nikon Co. Ltd., Tokyo Japan; Fig. 2, Fig. S1). The EMS is pre-installed with software for electric operation and control of electronic devices such as an electric injector via serial communication. The software also includes an image-processing function that automatically detects the position of the zygote (Fig. S2) and a sequence function that automatically moves the pipette to near the non-injected zygote or moves the injected zygote to the release area (Fig. S3). In the experiment for producing genetically modified mice, we used IAEMS, which was newly developed in LabVIEW 2017 for Windows (National Instruments, Austin, TX, USA) for fully automated insertion of the injection pipette into the pronucleus and integrated into the existing software. (Figs. 1, 3, 4, Movie S1). Fully automatic injection was performed under the solution injection conditions (injection time and injection pressure) experimentally derived for IAEMS. The solution injection in the control experiment was all performed manually using hardware that combined the EMS and electric injectors^11^.

All microinjections were performed using a holding pipette (outer diameter φ100 μm, inner diameter φ40 μm, bending angle 30°. HD052010040; Sankyo Medic Co., Ltd., Shizuoka, Japan) that holds the zygote and an injection pipette (tip angle 45°, inner diameter of the opening φ1 μm, bending angle 20°, and the volume of liquid in the pipette 3 μl. DNA-102001; Sankyo Medic) that injects the solution. The injection chamber was prepared by dropping 20 µL of M2 medium (M7167, Millipore Sigma, St. Louis, MO, USA) on a plastic dish (351,006, Corning Inc., Corning, NY, USA) and covering it with mineral oil (M8410, Millipore Sigma). The experiments were repeated using 15–30 zygotes for each solution infusion. Successful insertion of the injection pipette was confirmed by observing pronucleus swelling upon injection of the solution (Movie S1).

### Preparation of the injection solution for transgenic mice

For the traditional DI, the pEGFP-N1 vector (Clontech, Mountain View, CA, USA) containing the cytomegalovirus (CMV) promoter was digested with an AseI restriction enzyme (NEB, Ipswich, MA, USA) to generate a 4.7-kb linearized fragment (Fig. 6). The prepared fragment was diluted to 1.5 ng/µl with nuclease-free water (Promega, Madison, WI, USA) and injected into the pronucleus.

For piggyBac TS, piggyBac vector (1.33 ng/µl or 6.66 ng/µl) and transposase mRNA (0.66 ng/µl or 3.33 ng/µl) were dissolved in saline for total concentrations of 2 ng/µl and 10 ng/µl, respectively and injected into the pronucleus. The EGFP cDNA was cloned into the pENTR1A vector (A10462, Thermo Fisher Scientific Inc., Waltham, MA, US), and kindly provided by Dr. Takuji Maeda (Yokohama City University). The piggyBac vectors were constructed by LR reaction (11791-020, Thermo Fisher Scientific) of this entry vector and a PB-CA destination vector (circular, 7 kb; 20,960, Addgene, Watertown, MA, US) containing the CAG promoter (Fig. 6). The hyperactive piggyBac plasmid pCMV-hyperactive PBase was provided by the Medical Research Council^29^. This vector was linearized by digesting it with Age1, and the mRNA was synthesized by a mMESSAGE mMACHINE(tm) T7 ULTRA Transcription Kit (AM1345, Thermo Fisher Scientific). The mRNA was purified using a MEGAclear Kit (AM1908, Thermo Fisher Scientific).

### Preparation of the injection solution for knock-in mice

To generate knock-in mice, in which the P2A-EGFP sequence was inserted into the C-terminus of the Ddx4 gene, a targeting vector (Vasa-P2A-EGFP) was constructed (Fig. 6). The long arm was amplified by PCR from the mouse genome using a set of primers (Vasa5_S and Vasa5_AS), and P2A-EGFP was amplified by PCR from a synthetic P2A-EGFP template with a primer set (VasaP2A_S and VasaEGFP_AS). Using a mixture of these 2 amplified DNA fragments as the template, a PCR reaction was conducted with a set of primers (Vasa5PVU2_S and Vasa3EcoI_AS). The final product of this PCR reaction was P2A-EGFP flanked by ~ 90-bp short arm and a ~ 320-bp long arm with synonymous substitutions at the C-terminus of the Ddx4 gene. These substitutions were introduced to abolish re-targeting by Cas9-sgRNA to this locus, avoiding the introduction of mutations after the desired homologous recombination. The PCR product was digested using PvuII and EcoRI, and the digested DNA fragment was inserted into EcoRI and EcoRV sites of pLSODN-1 (DS615; BioDynamics Laboratory Inc., Tokyo, Japan) to generate the Vasa-P2A-EGFP targeting vector.

The single-stranded DNA (ssDNA) donor was prepared from the Vasa-P2A-EGFP targeting vector according to the manufacturer’s instructions for the Long ssDNA preparation kit (DS615; BioDynamics Laboratory). Briefly, the targeting vector was digested using EcoRI and nicking endonuclease, Nt. BspQ1. Formamide-based loading buffer (e.g., Denaturing Gel-loading Buffer from BioDynamics Laboratory) was added to the digested DNA. The mixture was heated to 70 °C to release the ssDNA, and then chilled on ice. The denatured DNA (200 µg) was loaded onto TAE-based non-denatured 1% agarose gels. The ssDNA was cut according to the single-stranded RNA marker and purified using NucleoSpin Gel and PCR cleanup (U0609B, TAKARA Bio Inc., Shiga, Japan).

For preparation of the guide RNA, crRNA and tracrRNA (Integrated DNA Technologies, Coralville, IA, USA) were annealed in Integrated DNA Technologies Duplex Buffer using a thermal cycler with a 1-µg concentration of the guide RNA. The guide RNA and Cas9 v3 protein (Integrated DNA Technologies) were assembled in water at room temperature (50 ng/µl each). Water and the ssDNA donor (final 2 ng/µl) was added to the guide RNA-Cas9 complex (final 20 ng/µl each). The solution was centrifuged for 30 min at maximum speed just before the injection. The supernatant was used for the injection.

To select a guide RNA with high performance, 3 different guide RNAs were tested (#1, #2, #3). A guide RNA-Cas9 complex and ssDNA were injected into the pronucleus of zygotes. When they reached the blastocyst stage (E3.5), DNA was extracted from each blastocyst using Quickextract DNA extraction solution (QE0905T; Lucigen Co. Ltd, Middleton, WI, US). PCR was performed using KOD ONE DNA polymerase (KMM-101 Toyobo Co. Ltd., Osaka, Japan) with a primer set (Vasageno_S and Vasageno_AS). The desired knock-in was most frequently observed when using guide RNA #3. We therefore used guide RNA #3 for the experiments.

### Analysis of transgenic mice

Traditional DI and piggyBac TS were analyzed by the same method. Expression of EGFP fluorescence in newborn transgenic mice was visualized by UV microscopy (VB-6000; KEYENCE Inc., Osaka, Japan) (Fig. 6). PCR genotyping of offspring was performed using a crude lysate of the tail tips (Fig. S4). Genomic DNA was extracted from the mouse tail using Direct PCR Lysis Reagent (Viagen Biotech Inc., Los Angeles, CA, US) containing 0.2 mg/ml proteinase K (Nacalai Tesque Inc., Kyoto Japan). The DNA lysate was used as a template in a 20-µl PCR reaction volume using go-Taq premixture (Promega Corporation) in the presence of the following primers to amplify the EGFP transgene and glyceraldehyde 3-phosphate dehydrogenase (GAPDH) as an internal control. EGFP forward 5′-GCGACGTAAACGGCCACAAG-3′, EGFP reverse; 5′-TAGGTCAGGGTGGTCACGAG-3′. GAPDH forward 5′-ACCACAGTCCATGCCATCAC-3′, GAPDH reverse; 5′-TCCACCACCCTGTTGCTGTA-3′.

### Analysis of knock-in mice

Because the EGFP protein is expressed in germ cells, the ovary or testis was irradiated with UV light and the resulting fluorescence was observed (SZX16; Olympus Co. Ltd., Tokyo Japan) (Fig. 6). PCR genotyping of offspring was performed using a crude lysate of the tail tips (Fig. S4). For PCR analysis, the same method as for guide RNA preparation was used.

### Statistical analysis

Experimental results of zygote survival and development into 2-cell stage embryos are expressed as mean ± standard error of the mean and statistical analysis was performed using the Student t-test after confirming a normal distribution. In all analyses, P < 0.01 was considered to indicate statistical significance. For analyses of the experimental data, Statcell 4 (OMS Publishing, Saitama, Japan), automated analysis software, was used.

## Supplementary Information

Supplementary Information 1.

Supplementary Video 1.

## References

[1] Kersten K, de Visser KE, van Miltenburg MH, Jonkers J (2017). Genetically engineered mouse models in oncology research and cancer medicine. EMBO Mol. Med..

[2] Davey RA, MacLean HE (2006). Current and future approaches using genetically modified mice in endocrine research. Am. J. Physiol. Endocrinol. Metab..

[3] Liggett SB (2004). Genetically modified mouse models for pharmacogenomic research. Nat. Rev. Genet..

[4] Babinet C (2000). Transgenic mice: an irreplaceable tool for the study of mammalian development and biology. J. Am. Soc. Nephrol..

[5] Gordon JW, Scangos GA, Plotkin DJ, Barbosa JA, Ruddle FH (1980). Genetic transformation of mouse embryos by microinjection of purified DNA. Proc. Natl. Acad. Sci. USA.

[6] Ivics Z (2009). Transposon-mediated genome manipulation in vertebrates. Nat. Methods.

[7] Largaespada DA (2009). Transposon mutagenesis in mice. Methods Mol. Biol..

[8] Meyer M, de Angelis MH, Wurst W, Kühn R (2010). Gene targeting by homologous recombination in mouse zygotes mediated by zinc-finger nucleases. Proc. Natl. Acad. Sci. USA.

[9] Wefers B (2013). Direct production of mouse disease models by embryo microinjection of TALENs and oligodeoxynucleotides. Proc. Natl. Acad. Sci. USA.

[10] Shen B (2013). Efficient knockin mouse generation by ssDNA oligonucleotides and zinc-finger nuclease assisted homologous recombination in zygotes. PLoS ONE.

[11] Behringer R, Gertsenstein M, Nagy A, Nagy K, Behringer R, Gertsenstein M, Nagy A, Nagy K (2014). Manipulating the Mouse Embryo: A Laboratory Manual. Fourth edition.

[12] Xu W (2019). Microinjection and Micromanipulation: A Historical Perspective. Methods Mol. Biol..

[13] Murphy D (1993). Microinjection of cloned DNA fragments into fertilized one-cell mouse eggs: II. Automatic injection. Methods Mol. Biol..

[14] Nagy A, Gertsenstein M, Vintersten K, Behringer R, Nagy A, Gertsenstein M, Vintersten K, Behringer R (2003). Manipulating the Mouse Embryo: A Laboratory Manual. Third edition.

[15] Pu XA, Young AP, Kubisch HM (2019). Production of Transgenic Mice by Pronuclear Microinjection. Methods Mol. Biol..

[16] Marh J (2012). Hyperactive self-inactivating piggyBac for transposase-enhanced pronuclear microinjection transgenesis. Proc. Natl. Acad. Sci. USA.

[17] Kaneko T, Mashimo T (2015). Simple genome editing of rodent intact embryos by electroporation. PLoS ONE.

[18] Ding S (2005). Efficient transposition of the piggyBac (PB) transposon in mammalian cells and mice. Cell.

[19] Giraldo P, Montoliu L (2001). Size matters: use of YACs, BACs and PACs in transgenic animals. Transgenic Res..

[20] Brinster RL, Chen HY, Trumbauer ME, Yagle MK, Palmiter RD (1985). Factors affecting the efficiency of introducing foreign DNA into mice by microinjecting eggs. Proc. Natl. Acad. Sci. USA.

[21] Wang W, Liu X, Gelinas D, Ciruna B, Sun Y (2007). A fully automated robotic system for microinjection of zebrafish embryos. PLoS ONE.

[22] Cornell E (2008). Automating fruit fly Drosophila embryo injection for high throughput transgenic studies. Rev. Sci. Instrum..

[23] Liu X (2011). Automated microinjection of recombinant BCL-X into mouse zygotes enhances embryo development. PLoS ONE.

[24] Yang H (2013). One-step generation of mice carrying reporter and conditional alleles by CRISPR/Cas-mediated genome engineering. Cell.

[25] Ballard DH (1981). Generalizing the Hough transform to detect arbitrary shapes. Pattern Recogn..

[26] Palermo G, Joris H, Devroey P, Van Steirteghem AC (1992). Pregnancies after intracytoplasmic injection of single spermatozoon into an oocyte. Lancet.

[27] Eto T, Takahashi R, Kamisako T (2015). Strain preservation of experimental animals: vitrification of two-cell stage embryos for multiple mouse strains. Cryobiology.

[28] Ho Y, Wigglesworth K, Eppig JJ, Schultz RM (1995). Preimplantation development of mouse embryos in KSOM: augmentation by amino acids and analysis of gene expression. Mol. Reprod. Dev..

[29] Yusa K, Zhou L, Li MA, Bradley A, Craig NL (2011). A hyperactive piggyBac transposase for mammalian applications. Proc. Natl. Acad. Sci. USA.

