# Supplementary material for: Establishment of an integrated automated embryonic manipulation system for producing genetically modified mice
Source: Sci Rep. 2021 Jun 3;11:11770. doi: 10.1038/s41598-021-91148-9 (PMC8175685; doi:10.1038/s41598-021-91148-9)
Supplement: Supplementary file 1 — Supplementary Information 1. [file 41598_2021_91148_MOESM1_ESM.pdf]

Supplementary Information

Title

Establishment of an integrated automated embryonic manipulation system for producing genetically modified mice.

Authors
*Tomoo Eto[1], Hiroki Ueda[2], Ryoji Ito[1], Tsukasa Takahashi[1], Toshiaki Watanabe[1], Motohito Goto[1], Yusuke Sotomaru[3], Nobuaki Tanaka[2], & Riichi Takahashi[1].

*Corresponding author: Tomoo Eto, etoh@ciea.or.jp

[1]Central Institute for Experimental Animals, 3-25-12, Tonomachi, Kawasaki-ku, Kawasaki, 210-0821, Japan
[2]New Field Products Development Center, NSK Ltd., 1-5-50, Kugenuma Shinmei Fujisawa-shi, Kanagawa 25-8501, Japan
[3]Natural Science Center for Basic Research and Development, Hiroshima University, 1-2-3, Kasumi, Hiroshima, Hiroshima, 734-8551, Japan

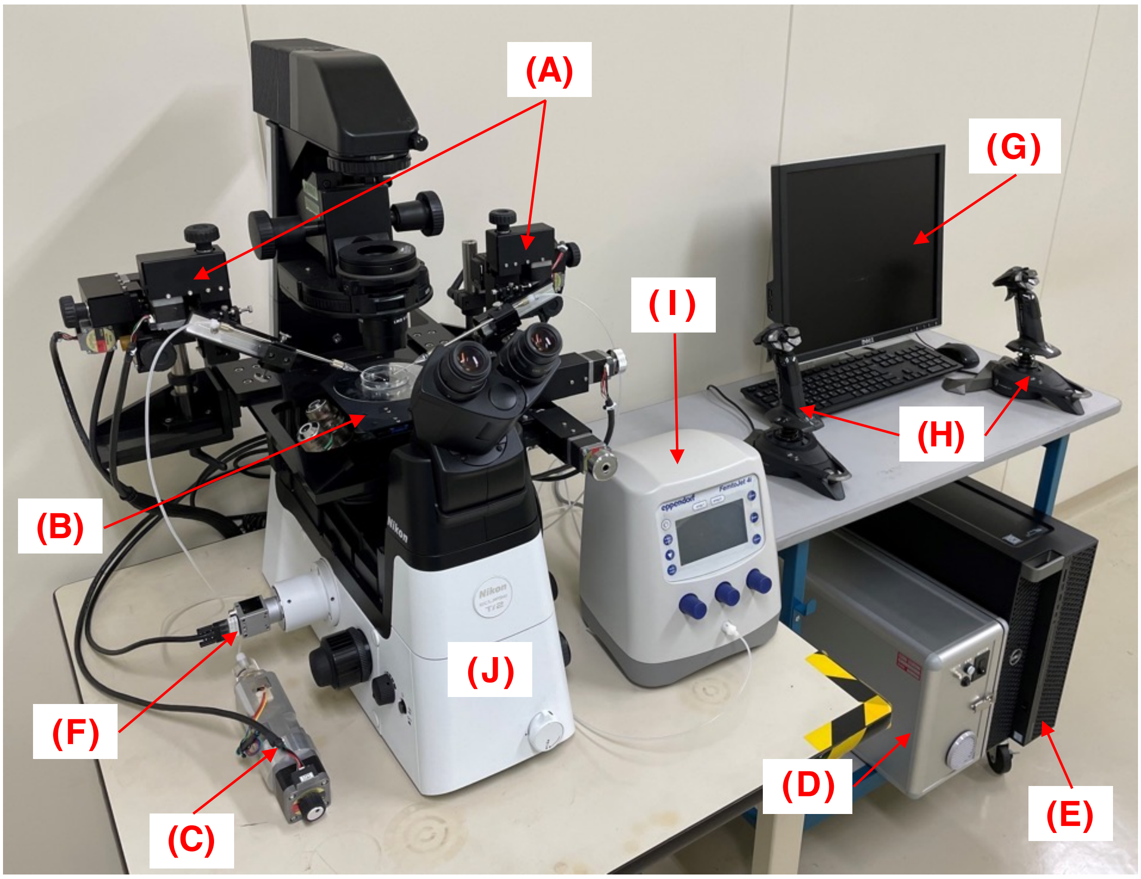

Figure. S1 Integrated Automated Embryo Manipulation System (IAEMS). The IAEMS consists of an EMS [(A) electric manipulator, (B) electric sample stage, (C) electric pump, electronic distribution board (D), controller (computer) (E), microscope camera (F), display (G), joystick(H)], electric injector (I), and microscope (J). LabVIEW software for Windows is installed on the controller, and is used to control each electronic device.

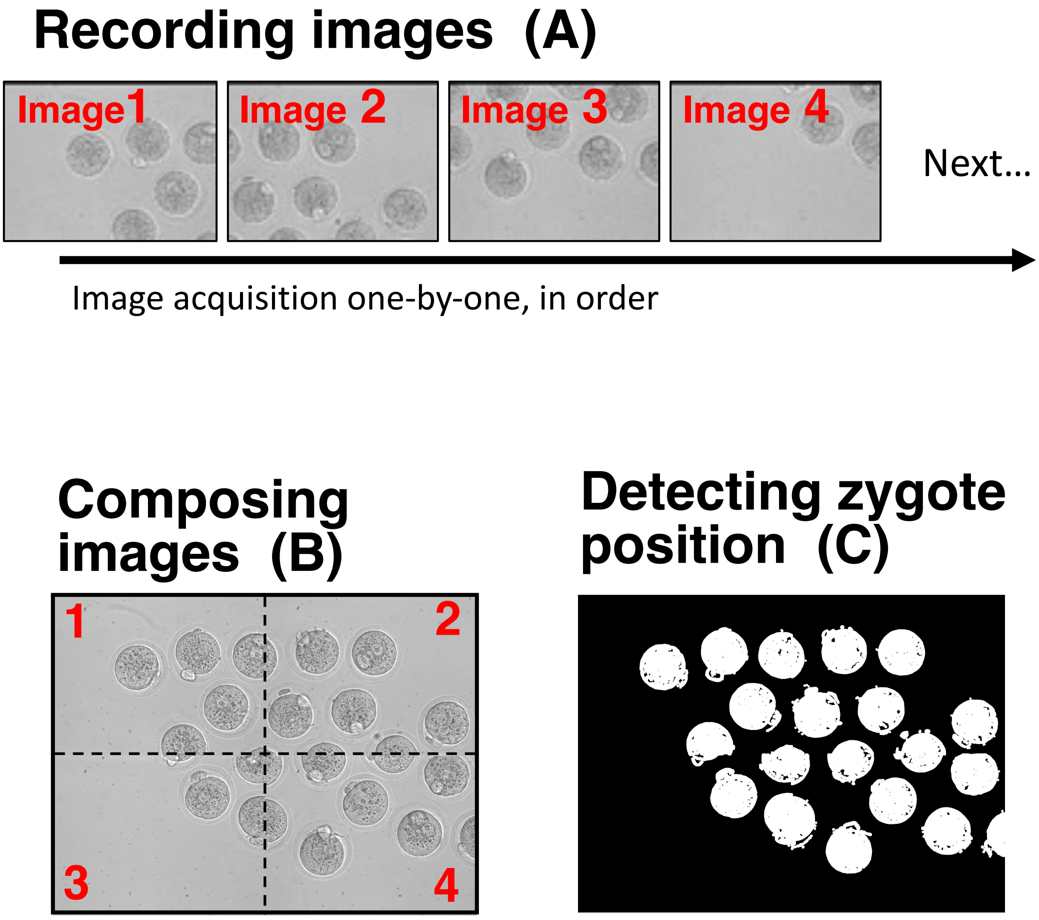

Figure. S2 Zygote position detection by the image processing function in the EMS software. (A) Record the microscopic images of the predefined area where the zygotes are placed in the order shown (red numbers). (B) Combine the acquired images to generate image data that enables detection over a wide area. (In this case, 4 images are combined). (C) Binarize the generated image and detect a particle object as a zygote.

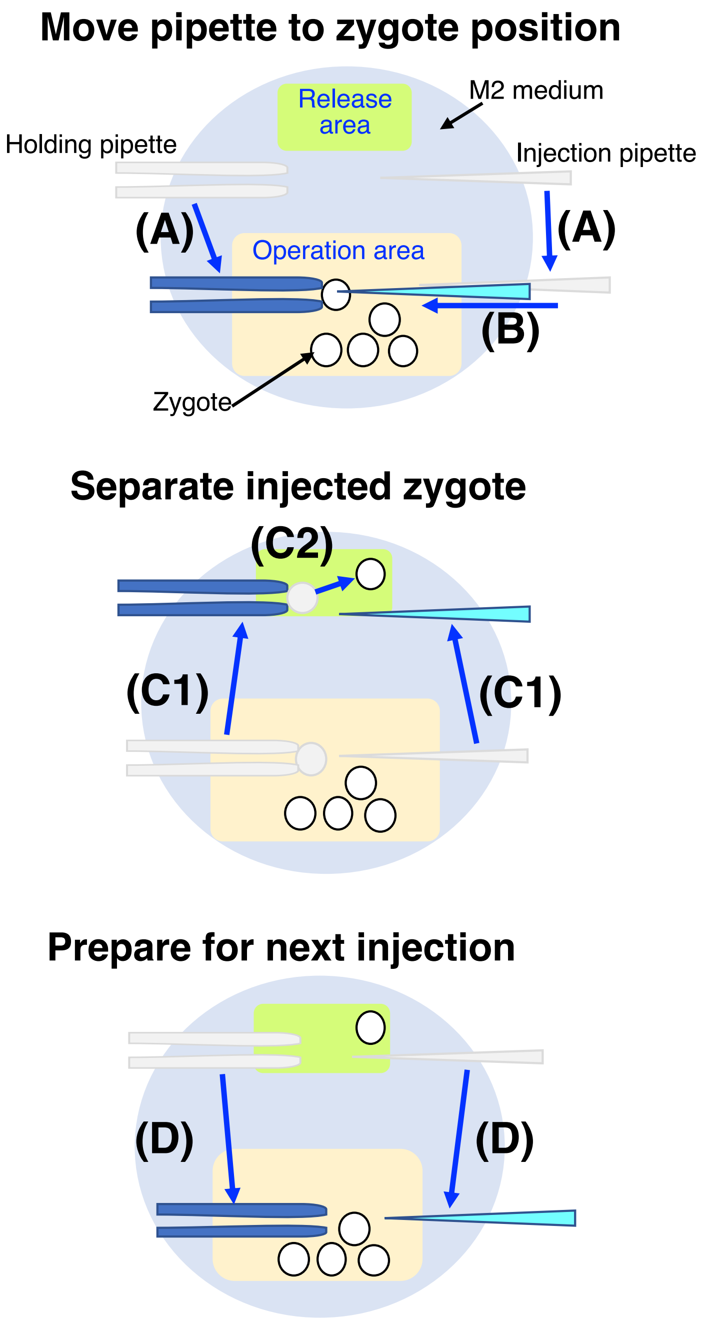

Figure. S3 Sequencing functions in the EMS software. The EMS can perform any sequence of operations by sending movement commands to the electric manipulators. This function can be used to move the pipette near the zygote, or to set up operation and release areas, and automatically move between each area to prevent mixing of injected and non-injected zygotes. (A) Move the pipette near to the non-injected zygote in the operation area based on detection of the zygote position. (B) Injection operation using the newly developed software. (C1,C2) While holding the injected zygote with the holding pipette, move to the release area 3000 µm away and release the injected zygote. (D) Return to the operation area and move the pipette to the next zygote to be injected.

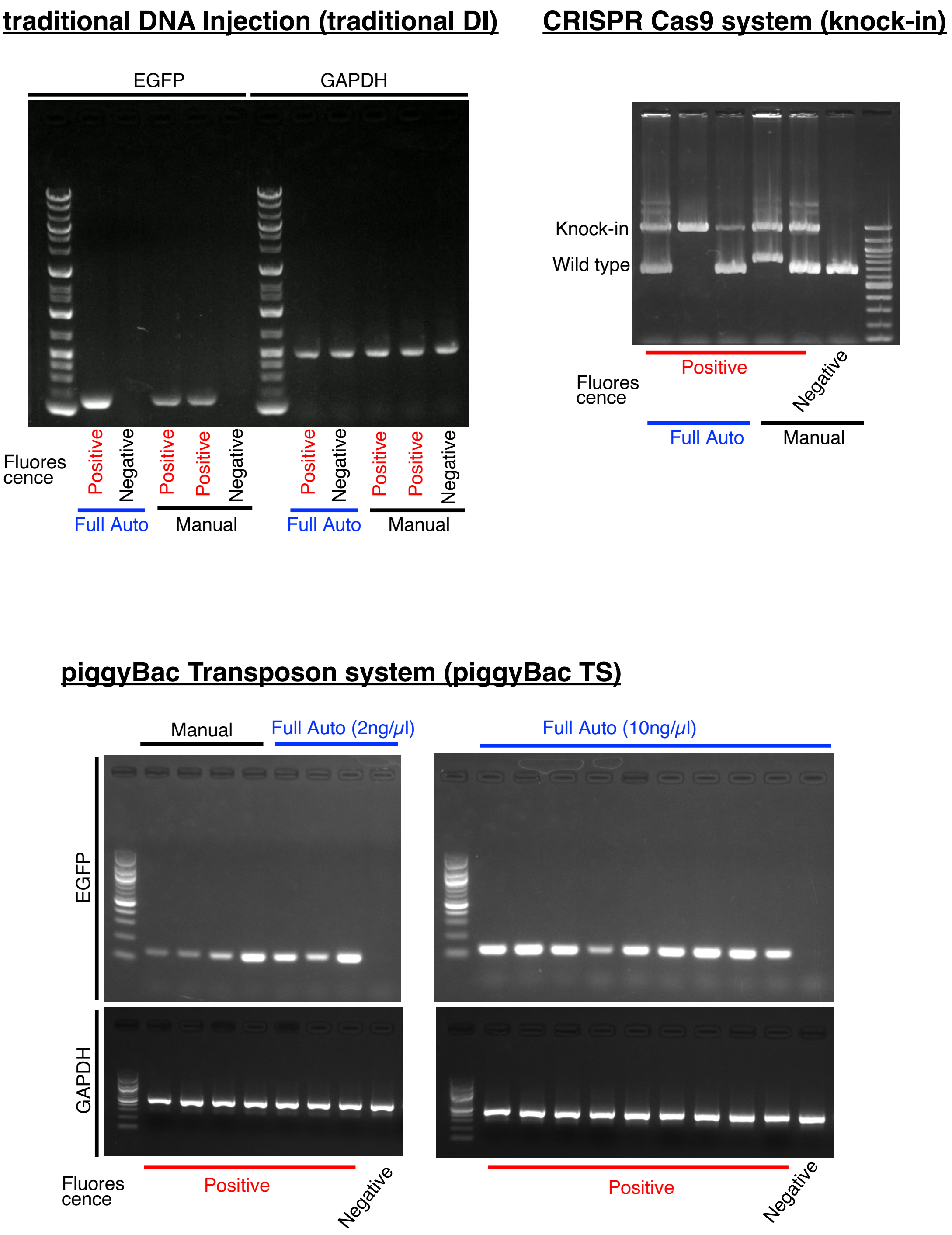

Figure S4. Results of genotyping analysis by PCR of genetically modified mouse DNA. This figure is a complement to cropped gels in the PCR analyses shown in Figure 6.

Specifications	Electric manipulator	Electric sample stage	Electric pump
Stroke	20 mm		1178.09 µl
Resolution	0.1µm		0.0039 µl/step
Maximum velocity	5 mm/s		5 rps
Driving method	5-phase stepping motor		

Table S1. Specifications of the electric manipulation system (EMS) device.

Genetic modification method	Injection method	Injection time (s)	DNA Concentration (ng/μl)	Number of pronuclear stage zygotes injected	Survival (% ± SEM)*	Developed to 2-cell stage embryo (% ± SEM)*
traditional DI	Full auto	0.8	1.5	151	132 (87.4 ± 2.4)	117 (88.6 ± 2.8)
		1.6	1.5	199	158 (79.4 ± 2.2)	141 (89.2 ± 1.9)
	Manual	-	1.5	148	128 (86.5 ± 2.6)	106 (82.8 ± 1.6)
piggyBac TS	Full auto	1.6	2	150	120 (80.0 ± 2.7)	110 (91.7 ± 1.7)
		1.6	10	150	124 (82.7 ± 1.3)	108 (87.1 ± 2.0)
	Manual	-	2	150	124 (82.7 ± 2.5)	114 (91.9 ± 2.0)
Knock-in	Full auto	1.6	2	199	161 (80.9 ± 3.7)	143 (88.8 ± 1.9)
	Manual	-	2	200	168 (84.0 ± 3.2)	135 (80.4 ± 2.8)

Table S2. Survival and *in vitro* development of zygotes injected into the pronucleus with solutions used for 3 different genetic modifications. *No significant difference was detected between experimental groups produced by the same genetic modification method.

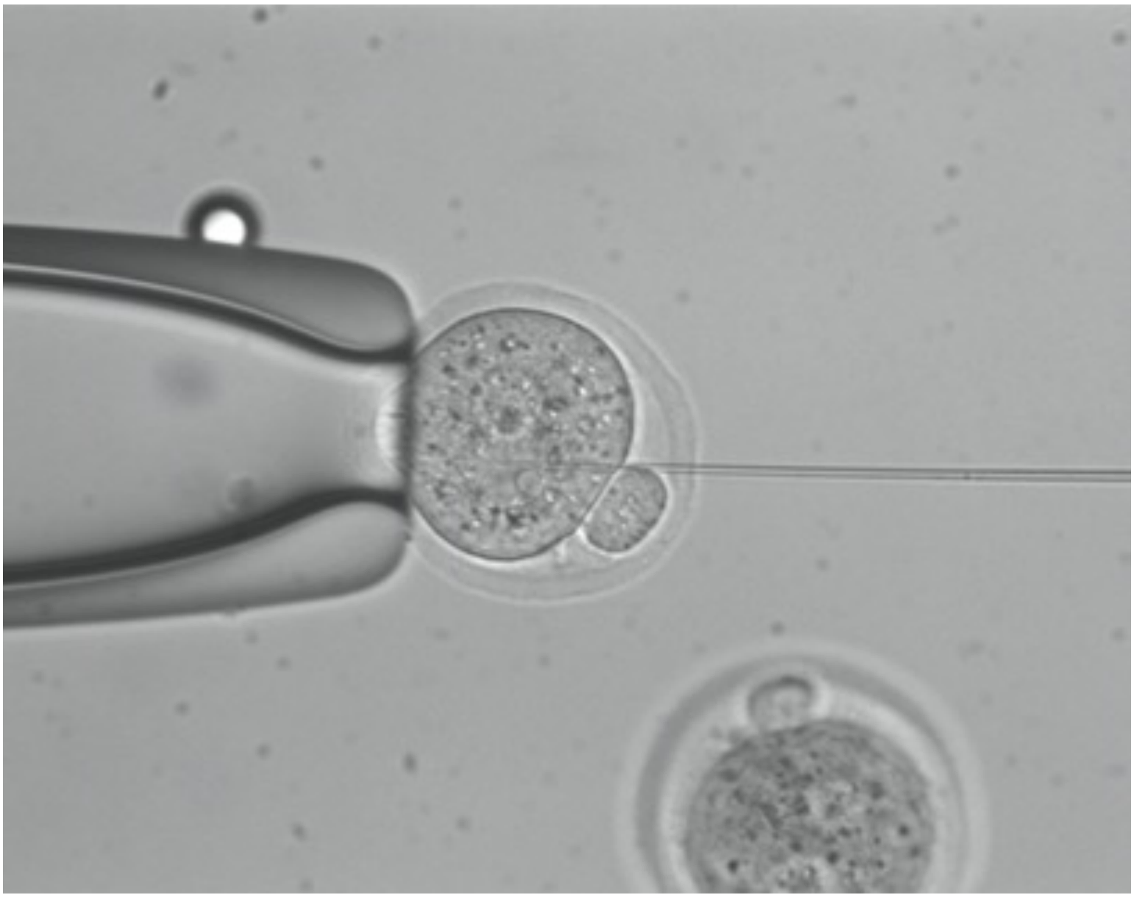

Movie S1 (separate file). Fully automatic injection into the zygote pronucleus.
